# Coding of attention across the human intraparietal sulcus

**DOI:** 10.1007/s00221-015-4507-2

**Published:** 2015-12-16

**Authors:** Jason D. Connolly, Robert W. Kentridge, Cristiana Cavina-Pratesi

**Affiliations:** Department of Psychology, Durham University Science Site, Durham University, Durham, DH1 3LE UK

**Keywords:** Intention, Attention, Functional magnetic resonance imaging, Posterior parietal cortex, Reaching, Eye movements

## Abstract

There has been concentrated debate over four decades as to whether or not the nonhuman primate parietal cortex codes for intention or attention. In nonhuman primates, certain studies report results consistent with an intentional role, whereas others provide support for coding of visual-spatial attention. Until now, no one has yet directly contrasted an established motor “intention” paradigm with a verified “attention” paradigm within the same protocol. This debate has continued in both the nonhuman primate and healthy human brain and is subsequently timely. We incorporated both paradigms across two distinct temporal epochs within a whole-parietal slow event-related human functional magnetic resonance imaging experiment. This enabled us to examine whether or not one paradigm proves more effective at driving the neural response across three intraparietal areas. As participants performed saccadic eye and/or pointing tasks, discrete event-related components with dissociable responses were elicited in distinct sub-regions of human parietal cortex. Critically, the posterior intraparietal area showed robust activity consistent with attention (no intention planning). The most contentious area in the literature, the middle intraparietal area produced activation patterns that further reinforce attention coding in human parietal cortex. Finally, the anterior intraparietal area showed the same pattern. Therefore, distributed coding of attention is relatively more pronounced across the two computations within human parietal cortex.

## Introduction

There has been a vast amount of work in nonhuman primates (Andersen 1995; Andersen and Buneo 2002; Andersen et al. 2010; Bisley and Goldberg 2003, 2010; Bushnell et al. 1981; Colby and Goldberg 1999; Cui and Andersen 2011; Ganguli et al. 2008; Goldberg and Bushnell 1981; Mountcastle et al. 1975; Quian Quiroga et al. 2006; Snyder et al. 1997, 2000), and now humans (Astle et al. 2012; Connolly et al. 2003; Levy et al. 2007), that has investigated the role of intention or attention in the parietal cortex. Here we define intention as early motor planning and attention as a visuospatial shift in locus in the absence of early motor planning or of any overt movement planning whatsoever. Although certain studies and subsequent reviews have emphasized the role of the nonhuman primate PPC in intention (Andersen 1995; Andersen and Buneo 2002; Cui and Andersen 2011; Mountcastle et al. 1975; Quian Quiroga et al. 2006; Snyder et al. 1997, 2000), others have stressed a relatively greater role in visuospatial attention (Bisley and Goldberg 2003, 2010; Bushnell et al. 1981; Colby and Goldberg 1999; Ganguli et al. 2008; Goldberg and Bushnell 1981). It could be the case that the particular paradigm employed by these different camps induces an intentional or attention bias in the underlying neuronal activity. We therefore combined two types of verified paradigms (Bisley and Goldberg 2003; Snyder et al. 1997) within the same experimental protocol, such that we could examine whether or not intention (Snyder et al. 1997) or attention (Bisley and Goldberg 2003) coding is relatively more dominant, and if so, where. Using a slow event-related experimental approach via functional magnetic resonance imaging (fMRI), we report evidence for distributed coding of attention in the human intraparietal sulcus. Critically, this approach allowed us to successfully integrate these two confirmed paradigms from the nonhuman primate literature in one experimental design and to examine the results throughout the entire intraparietal sulcus (IPS) simultaneously. The protocol involved presenting both a double dissociation task—in which the participant was required to anticipate making simultaneous saccade and pointing movements in opposite directions (Snyder et al. 1997) (an intention coding paradigm) and then were subsequently instructed to further anticipate or cancel this very same anticipation (Bisley and Goldberg 2003) (an attention coding paradigm). Using this approach, we demonstrate in the healthy human brain that areas within the IPS code relatively preferentially for visuospatial attention.

Participants were first shown cues where possible future simultaneous saccadic eye movement/pointing movements should be directed and subsequently were shown two probe cues that instructed which—if any—responses were to then be required. They were then instructed to either execute and/or withhold these very same movement responses. This paradigm gave rise to three distinct event-related epochs: intention, attention and motor activity/response suppression. Upon examining these functional MRI time courses, we were able to then individually assess the extent to which the different PPC areas are enmeshed in intention or attention.

We predicted that intention would drive the most posterior portion of the IPS (or pIPS). In other words, the pointing trials would show greater fMRI-BOLD activation than saccade trials particularly during Epoch 1. On the other hand, attention would drive the middle portion of the IPS (or mIPS). In other words, there would be no difference between eye and hand fMRI-BOLD activity profiles during Epoch 1 in this region. Finally, based on its known involvement in reaching and grasping, we predicted that the anterior IPS (or aIPS) would have activity that would be higher for the hand over the eye during the intention phase (or Epoch 1), similar to posterior IPS. However, here we report that all three regions showed no difference and therefore must—at least preferentially—code for visuospatial attention, vis-à-vis none of the IPS zones showed preferential coding for the hand over the eye (or vice versa). In other words, epoch activity overlapped for eye and hand in all three regions. Critically, all three of the IPS zones showed similar levels of activity during Epoch 2, irrespective of whether or not the movement was cancelled. This observation further reinforces that attention-based coding relatively predominates throughout the human IPS and this experimental finding is consistent with certain earlier studies in the literature.

## Methods

### Participants

Six neurologically intact male participants with normal or corrected-to-normal vision (age range 23–56) each participated in two functional MRI scanning sessions, for a total of 11 sessions [one scan session had to be discarded for one participant owing to a lack of activation within particular regions of interest (ROIs)]. All participants provided written informed consent and used their right hand to execute the “rotation-about-the-wrist” pointing movements. The study was approved by the Newcastle University Faculty of Medical Sciences Ethics Committee.

### Visual stimuli and task

**Fig. 1:**
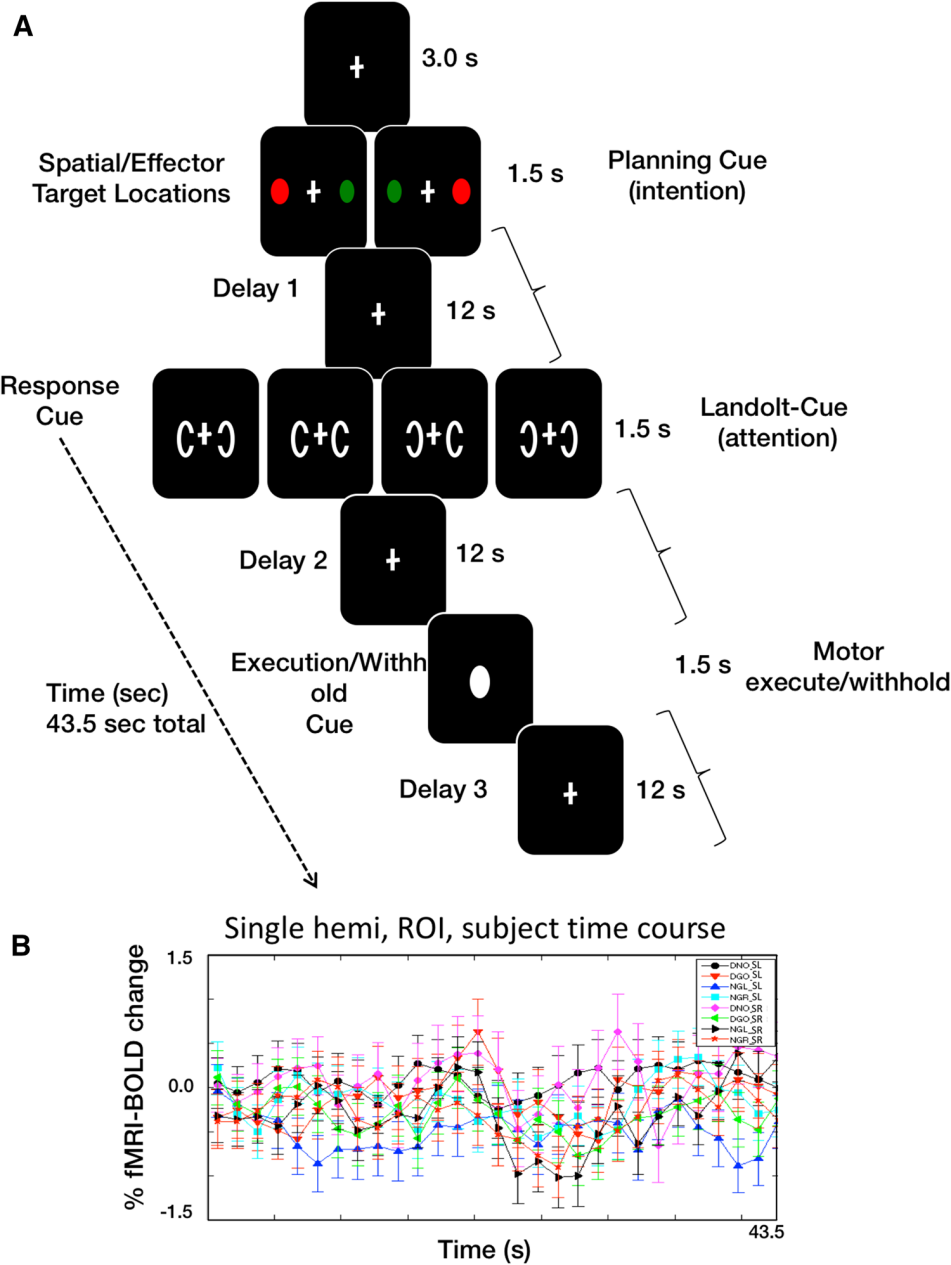
The experimental protocol for dissociating intention and attention by PPC brain area. **a** Following a fixation baseline interval, *two circles* are flashed simultaneously and at the same eccentricity but on opposing sides of the *horizontal* meridian. *One circle* is *green* (instructing the participant to prepare for the possibility of a saccadic eye movement response later in the trial) and the other *red* (instructing the participant to prepare for the possibility of a pointing response later in the trial). The *colour* and the side of *each circle* indicated to the participants which effector (eye or hand) to use and in which visual hemifield (intention cue, or Epoch 1). This is then followed by a 12-s “cueing phase”. Then, one of four combinations of C-shaped Landolt probes are presented at a constant eccentricity that is smaller than that of the previous *red*/*green* cues (attention cues). For each of these C-shaped probes, a gap oriented towards the centre of the screen indicated that the participant is to continue to intend to generate the movement type previously indicated on that side (either saccade or point), whereas a gap oriented away from fixation instructed the participant to abolish the intent (no-go trial). Participants therefore are then required to: (1) generate a simultaneous saccade/point (in opposite directions), (2) either saccade or point or (3) withhold both movements (double no-go). The Landolt probes are followed by a 12-s motor preparation phase. Subsequently, a *white circle* appeared at fixation that instructed the participant to execute or withhold the movement(s) specified by the preceding cues (execution/withhold cue). **b** A randomly selected “raw” time series for a single participant, hemisphere and ROI. It can be seen that there are three peaks in the fMRI-BOLD responses and these correspond to our three functional epochs of interest

Participants maintained central fixation for the first 3 s of each trial, followed by a 1.5-s simultaneous presentation of a red and a green circle (“intention” cues) (Snyder et al. 1997) along the horizontal meridian on opposite sides of fixation and at equal eccentricity (on every trial, both cues were presented at equal eccentricities that randomly varied between 6° and 9°). There were two possible pairs of cues, either green left and red right or red right and green left (Fig. 1). The red circle indicated the location of a possible future pointing movement (and the subsequent movement was to be withheld on 50 % of the trials, based on the upcoming Landolt probe cues), and the green target indicated the location of a possible future saccade (which was also to be withheld on 50 % of the trials). The circle cues were then followed by a 12-s interval in which the participant held central fixation and remembered the specific effector associated with each side of fixation (intentional epoch, or Epoch 1). For Epoch 1, given that the green cue represented a saccade in one direction and a pointing movement in the opposite direction, then—provided that the event-related analyses are separated by hemisphere—it was then possible to examine whether or not there was movement planning activity dedicated to a particular effector.

This first cueing interval was then followed by two simultaneous go or no-go “Landolt C’s” or “attention” cues (Bisley and Goldberg 2003) on opposite sides of the horizontal meridian (always at 5° eccentricity, which was different than the eccentricity of the red/green circle target cues). Go cues contained a gap that was oriented towards the central fixation point, while no-go cues had a gap oriented away from fixation (Fig. 1). Epoch 2 is designed such that if attention coding is present, then there should be no attenuation of the fMRI-BOLD response—or the signal should remain elevated—even when the movement is entirely cancelled on 50 % of all trials. Alternatively, if such activation represents motor planning activity, then on the cancelled trials, the signal should decay towards baseline. There were four possible cue combinations: (1) double go (DGO), (2) double no-go (DNO), (3) go left/no-go right (NGR) and (4) no-go left/go right (NGL). There were therefore a total of eight trial types (two for green/red location by four go/no-go probe cue configurations). The green cue represented an instruction to plan a saccade in that direction and to plan a pointing movement towards the red cue. So, in the figures, the final two letters indicate the location of the saccade target (SL—saccade left-side target or SR—saccade right-side target). To provide an example, DGOSL refers to “DGO—saccade left and point right”.

Presentation of the Landolt probe cues was followed by a 12-s motor preparation interval. The fixation spot was replaced by a circle for 1.5 s, and this instructed the participant to execute or withhold the saccade and/or pointing movement(s) specified by the preceding cues. For example, a trial with the green cue on the left and the red on the right followed by a DGO cue would instruct the participant to make a saccade to the left and a simultaneous pointing movement to the right during the motor phase of the trial (at the outset of Epoch 3). Participants executed pointing movements by rotating the right hand about the wrist. Movement execution/withholding was thus followed by a final motor interval of 12 s, which provided sufficient time for the functional MRI signal to return to baseline (Fig. 1).

### Stimuli presentation and data collection

A Canon XEED LCD projector was used to project the visual stimuli onto a screen that was viewed through an angled mirror attached to the head coil. Stimulus presentation was controlled with the Psychophysics Toolbox (www.psychtoolbox.org). Eye and hand movements were directed towards the presented stimuli and monitored online using an MR-compatible eye tracker (Applied Science Laboratories, Bedford, MA, USA) located at the back of the scanner (near the head coil) and an MR-compatible camera (MRC Systems GmbH, Heidelberg, Germany) that was positioned at the participant’s feet and was used to record the pointing movements online. Eye (leftward/rightward saccades) and hand (leftward/rightward wrist rotations) movements were directed towards the left and the right stimuli in an ecological manner as possible following two 15-min training sessions prior to fMRI scanning. Whole-arm reaching was avoided to reduce motion translation to the shoulder and subsequent head motion. Errors were thus *extremely* rare (indeed, the functional run was halted and the scan discarded if a behavioural error occurred during a particular scan run and this occurred for 6 % of all scan runs collected). These recordings were used to verify online—or in the actual scanner control room—that each of the participants was performing the tasks correctly and consistently across all trials types or conditions of interest for a particular functional scan run. We viewed the eye movement directions and hand pointing directions via two separate video monitors and two experimenters (J.D.C. and C.C.P. were necessary to accomplish this—or one person monitoring a particular effector movement or video monitor during the experimental protocol). So, only entirely correct functional runs were included in the subsequent analysis. Moreover, there were <2 functional runs discarded per participant using this straightforward procedure.

### Functional imaging

Functional data were acquired using a Philips eight-channel receive-only SENSE head coil on a 3-T scanner (Philips Intera Achieva) at the Newcastle Magnetic Resonance Centre. For each functional scan, a $T_{2}^{*}$-weighted echo-planar image (EPI) pulse sequence was employed (TR: 1500 ms, TE: 30 ms, flip angle: 75°, 30 slices, 3 × 3 × 3 mm voxels, FOV: 192 mm). Data were collected for 30 coronal slices that covered the entire parietal cortex and extended anteriorly to the back of the frontal lobe. Four scans were collected prior to the onset of each functional scan run to eliminate the transient effects of magnetic saturation. Ten functional runs were collected per participant per session. In each functional run, there was one repetition of each of the eight trial types (or eight trials per functional run). There were therefore 20 repetitions across the two sessions per participant of each trial type (or for each of the eight conditions in our 2 × 4 factorial design). Therefore, in a particular testing session, there were 80 trials collected using this slow event-related approach. In the average time courses (Fig. 3), this amounts to 220 trials per condition. Each of the conditions had an initial baseline state of 15 s in which the participant was required to look at central fixation with the hand resting on their upper abdomen.

### Preprocessing and analysis

The data were high-pass filtered at a cut-off frequency of 0.01 Hz at each voxel to remove the slow drift of functional MRI data. Then the time series for each voxel was shifted in time by 5 s to compensate for the hemodynamic lag.

Visualizations were based on segmenting the grey and the white matter in the *T*_1_-weighted scans. We used FreeSurfer (http://surfer.nmr/mgh.harvard.edu/fswiki) to accomplish this computationally. Functional MRI data were analysed using mrTools MATLAB-based software from the Heeger Lab [New York University: (http://www.cns.nyu.edu/heegerlab/)], and subsequent analyses (Fig. 3) were carried out using our own custom MATLAB (MathWorks, MA, USA) code. We used a combination of FreeSurfer and finally SurfRelax (http://www.pc.rhul.ac.uk/staff/J.Larsson/software.html) to import the surfaces and to generate the flat maps within the mrTools environment. This was done in order to restrict the functional data analyses to grey matter voxels only. We then inflated the cortical surfaces and computed and displayed the *R*^2^ activation maps on flattened surface maps of the parietal cortex, and these were also computed using the mrTools software.

### *R*^2^ functional maps

All functional scans for a particular participant were concatenated together into one single long functional run using the mrTools software. This enabled us to convert to % fMRI-BOLD signal change and to generate one long scan such that we could estimate the responses using all the data—or to analyse data that were collected over many consecutive scans. Moreover, this enabled the high-pass filtering to be done on a single data set for each participant.

Motion compensation was carried out using the mrTools software. We used a cubic interpolation method with three iterations (http://gru.stanford.edu/doku.php). The timing of the events in the experiment was used for the event-related analysis as follows: we over-paramaterized our event-related model, such that every functional volume was deemed to be a factor, and thus, every functional volume received a value of “1”. This allowed the extracted event-related time series to adhere to any possible waveform shape (or the time series could have any number of possible signal peaks or modulations possible; however, refer to Fig. 1b which shows three clear modulations that correspond to each of our three epochs of interest). Moreover, each and every one in the averaged time series of 29 averaged volumes per condition then becomes naturally scaled to the amplitude of the time series. This approach has been argued to represent the correct scale for per cent signal change: (http://gru.stanford.edu/doku.php).

The *R*^2^ maps informed us as to what proportion of the variance in each time course is accounted for by the average hemodynamic response. The *R*^2^ maps each had a cut-off of 0.22 or the model accounted for at least 22.0 % of the variance—or even exceeded this cut-off threshold—for all of the individual sessions. This value was used for visualization purposes only or to “clean-up” the noise in the maps for the subsequent slow event-related analyses. Travelling wave standard error bars were then plotted over the averaged event-related time series of interest using MATLAB code (MathWorks, MA, USA). These were computed as the residual variance distributed back to each individual time point.

### ROI definition of the IPS zones on the flat maps

We drew the ROIs using the mrTools software (http://gru.standford.edu/doku.php) and on the actual grey matter flat maps. We plotted anchor points along key anatomical landmarks within the posterior parietal cortex. We first plotted an anterior anchor point (at the precise junction of the IPS and the post-central sulcus) on the inflated maps. We then drew a posterior anchor point (at the junction of the IPS and the parieto-occipital sulcus) on the inflated maps. These two anchor points always remained visible on the flattened surfaces, and we were able to alternate back and forth between the inflated surface and the flat maps to ensure that these were both placed accurately. The flattened surfaces subsequently show a posterior (or “back” anchor point) and an anterior (or “front”) anchor point. We then partitioned the IPS based upon these same two anchor points into three equal-sized zones on the flat maps, which we refer to as posterior IPS, middle IPS and aIPS (refer to “Results” section). In order to draw these ROIs as precisely as possible, we drew square (or rectangular) individual ROIs of equal size for each of the three IPS zones and this procedure ensured that each of the ROIs was of the same size on the flattened surface along the above-noted extent of the IPS—yet also allowing for the fundus of the IPS to be positioned at the middle of each of these same ROI drawings. Each ROI therefore included ~equal portions of both the inferior PPC and superior PPC voxels and to an equivalent degree as was possible via manual drawing. Once these had been drawn for all 12 scanning sessions, we then extracted the time series for each and every ROI separately. The ROIs were thus calculated individually for each and every participant for each hemisphere and for each experimental condition in our 2 × 4 Factorial experimental design.

**Fig. 2:**
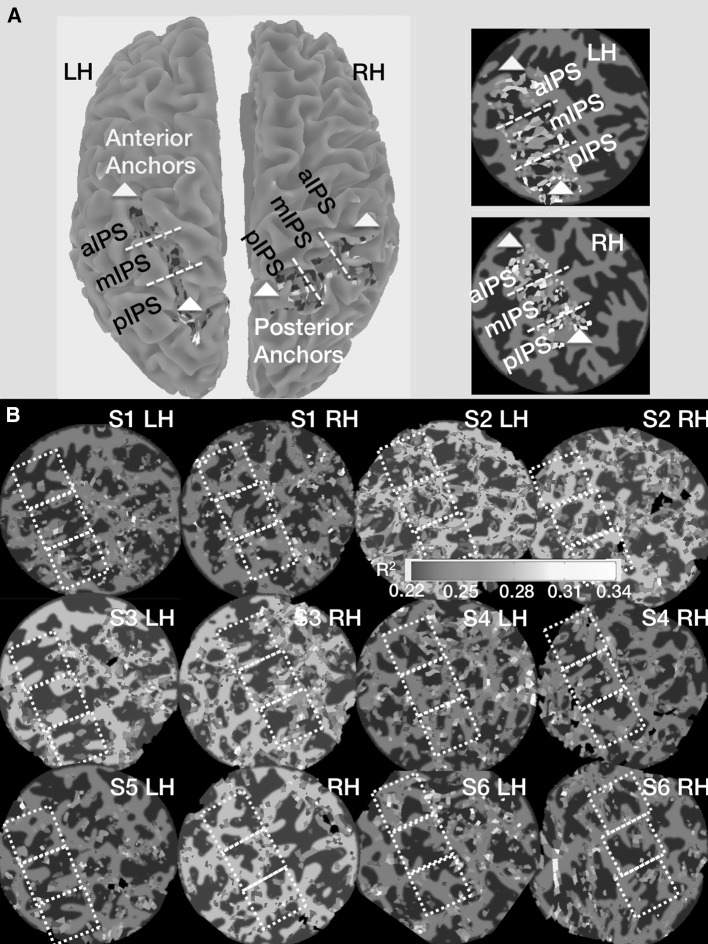
GLM-based maps show the ROI location on the surface and threshold variance-explained (*R* ^2^) maps for single scan sessions. **a** The dorsal surface of the left (LH) and right (RH) cerebral hemispheres. We first used the mrTools software GLM-based analysis that allows us to selectively show activation within our three regions of interest (ROIs) (see “Methods” section). This is used to ascertain that ROIs included the IPS and is distinct from the slow event-related analysis. It is clear from this representative drawing that our ROIs captured the full extent of the IPS, from the most posterior point (*white anchor point*) at the junction of the parieto-occipital and IP sulci to the most anterior IPS at the junction of the post-central and IPS (*yellow anchor point*). The flat maps to the *right* side of the surface map in **a** shows the equidistant parsing procedure used. Briefly, we drew *two lines* that partitioned the IPS based on the *above anchor points* into three equal-sized zones from back to front (pIPS, mIPS and aIPS, respectively). **b** The data from the single scan session displayed on each participant’s flattened surface representation (*R* ^2^ > 0.22 or at least 22 % of the variance is accounted for by the model). As can be seen, there is activation distributed along both the inferior (BA7) and superior (BA5) portions of the parietal lobule. Although 11 scan sessions are included, we only show one session for each participant (although all but one of the 12 sessions collected demonstrated activation in each and every ROI)

We further calculated a direct GLM model for visualization purposes only (Fig. 2a) to ensure our drawings was accurate. The GLM approach as implemented in mrTools (http://gru.standford.edu/doku.php) was simply used for visualization purposes, as this provides for the anatomical localization of the activation *within the ROIs only*—and therefore *excludes activation outside the ROIs*. This enabled us to confirm that our ROI drawings were in highly consistent alignment with the IPS (Fig. 2a). Finally, when calculating the signal peaks, we used a “rolling-maximum” calculation within each of the epochs via custom MATLAB code (MathWorks, MA, USA). This allowed us to calculate the fMRI-BOLD signal peaks within each of the three epochs (intention, attention, and movement execution/withhold) of interest. These were identified via MATLAB parsing the event-related time series into three separate epochs and then determining the peak signal amplitude within each epoch. Within each of these three epochs, we calculated the peak fMRI-BOLD response for each condition (out of eight conditions in total) that was further separated by hemisphere and the three IPS ROIs.

For the final statistical analyses, mean values were calculated for each participant for all dependent measures collapsed across all replications for each possible combination of hemisphere, epoch, condition and IPS zone (or ROI). These mean values were then entered into a separate 2 × 3 × 8 × 3 (hemisphere × epoch × condition × ROI) repeated-measure analysis of variance (ANOVA).

## Results

We conducted a Fourier transform of the raw single participant, single hemisphere and single region functional MRI responses to determine the number of signal peaks in the time courses. There was a pronounced peak for a randomly selected and representative time series: S1 (participant), right hemisphere (RH) and anterior intraparietal area (aIPS) at 0.0685 Hz (or ~3 cycles per averaged time series). This same pattern was observed across all participants, both hemispheres and in all IPS zones. This gives us confidence that the data exhibited three peaks corresponded to the three unique functional epochs (Fig. 1a) of our experimental protocol. Figure 1b shows a representative left hemisphere mIPS time series, and it is clear that there exist three separate signal peaks.

As noted in the “Methods” section, we first used the GLM procedure in the mrTools software to show that the activation in each of the ROIs was localized to the IPS. However, a slow event-related design was best suited for the present experimental design and was used here. The GLM analysis, however, allowed us to show the activation with the software within the ROIs only and thus excluded all other grey matter activation. As shown in Fig. 2a, the three ROIs—as drawn on the flattened grey matter only maps (posterior, middle and aIPS)—corresponded extremely well with the entire posterior–anterior extent of the IPS and all ROIs were centred on the fundus on the actual inflated surface maps. We then proceeded to follow the very same drawing procedure for all of the other scanning sessions. As shown in Fig. 2b, there was activation throughout the lateral (Brodmann area 7) and medial (Brodmann area 5) area of the parietal lobe using the IPS as the key lateral/medial landmark. However, we restricted our analyses to these three equal-sized IPS ROIs that included and extended along the fundus of the IPS and included grey matter that extended ~equidistantly both medial (BA 5) and lateral (BA 7) to the fundus.

Figure 2b shows that there was activation in each of our ROIs with the exception of one session for one participant (one session excluded from further analyses out of all 12 scan sessions, resulting in 11 sessions total), and this enabled us to extract the event-related time series from all ROIs and on an individual fMRI session-by-session basis. Again, one session had to be excluded from this analysis owing to an absence of activation in one of our IPS ROIs. As noted in the “Methods” section, the signal peaks were extracted for each of the three epochs of interest using a rolling-maximum calculation and the signal peaks and standard deviations separated by hemisphere are reported in Tables 1, 2 and 3 (these tables correspond to pIPS, mIPS and aIPS, respectively). What is notable with regard to these tables is that the means portray such an extreme degree of “overlap” or consistency in the signal amplitudes for all epochs, all conditions, all IPS zones and across both hemispheres that were examined in the present study.

**Table 1:** Mean signal peaks and standard deviations for each of the two hemispheres and three epochs sorted by condition for pIPS

	Left hemi			Right hemi		
	1	2	3	1	2	3
NGR SR	0.43 (0.24)	0.42 (0.22)	0.42 (0.24)	0.41 (0.54)	0.36 (0.27)	0.50 (0.44)
NGL SR	0.36 (0.22)	0.34 (0.18)	0.32 (0.18)	0.54 (0.32)	0.50 (0.54)	0.61 (0.59)
DGO SR	0.40 (0.23)	0.34 (0.22)	0.34 (0.18)	0.37 (0.32)	0.35 (0.41)	0.35 (0.29)
DNO SR	0.38 (0.19)	0.40 (0.24)	0.37 (0.22)	0.56 (0.36)	0.48 (0.37)	0.49 (0.32)
NGR SL	0.45 (0.25)	0.38 (0.18)	0.37 (0.17)	0.47 (0.28)	0.42 (0.39)	0.49 (0.49)
NGL SL	0.37 (0.22)	0.37 (0.15)	0.34 (0.14)	0.42 (0.28)	0.31 (0.30)	0.43 (0.31)
DGO SL	0.40 (0.26)	0.40 (0.17)	0.39 (0.24)	0.43 (0.39)	0.48 (0.42)	0.58 (0.46)
DNO SL	0.40 (0.28)	0.36 (0.19)	0.35 (0.20)	0.53 (0.40)	0.45 (0.50)	0.42 (0.36)

**Table 2:** Mean signal peaks and standard deviations for each of the two hemispheres and three epochs sorted by condition for mIPS

	Left hemi			Right hemi		
	1	2	3	1	2	3
NGR SR	0.44 (0.33)	0.39 (0.21)	0.34 (0.20)	0.40 (0.26)	0.40 (0.32)	0.37 (0.23)
NGL SR	0.42 (0.31)	0.34 (0.24)	0.38 (0.22)	0.40 (0.27)	0.36 (0.18)	0.33 (0.31)
DGO SR	0.32 (0.25)	0.32 (0.25)	0.37 (0.29)	0.35 (0.17)	0.38 (0.25)	0.31 (0.26)
DNO SR	0.41 (0.34)	0.40 (0.21)	0.37 (0.34)	0.48 (0.37)	0.51 (0.41)	0.37 (0.30)
NGR SL	0.43 (0.31)	0.42 (0.28)	0.34 (0.16)	0.36 (0.20)	0.32 (0.21)	0.31 (0.25)
NGL SL	0.34 (0.34)	0.35 (0.23)	0.35 (0.25)	0.33 (0.16)	0.35 (0.17)	0.32 (0.30)
DGO SL	0.44 (0.36)	0.43 (0.23)	0.37 (0.18)	0.38 (0.23)	0.39 (0.22)	0.31 (0.19)
DNO SL	0.37 (0.30)	0.44 (0.45)	0.34 (0.20)	0.38 (0.23)	0.34 (0.20)	0.33 (0.21)

**Table 3:** Mean signal peaks and standard deviations for each of the two hemispheres and three epochs sorted by condition for aIPS

	Left hemi			Right hemi		
	1	2	3	1	2	3
NGR SR	0.42 (0.33)	0.39 (0.24)	0.53 (0.51)	0.37 (0.31)	0.43 (0.28)	0.50 (0.21)
NGL SR	0.46 (0.45)	0.33 (0.20)	0.43 (0.41)	0.38 (0.20)	0.39 (0.19)	0.45 (0.22)
DGO SR	0.40 (0.32)	0.28 (0.17)	0.48 (0.40)	0.39 (0.18)	0.44 (0.33)	0.36 (0.29)
DNO SR	0.45 (0.37)	0.40 (0.37)	0.46 (0.41)	0.49 (0.40)	0.51 (0.33)	0.37 (0.28)
NGR SL	0.39 (0.29)	0.36 (0.27)	0.46 (0.37)	0.39 (0.23)	0.34 (0.23)	0.36 (0.23)
NGL SL	0.32 (0.21)	0.40 (0.19)	0.41 (0.40)	0.36 (0.20)	0.43 (0.34)	0.35 (0.23)
DGO SL	0.42 (0.37)	0.37 (0.27)	0.52 (0.52)	0.46 (0.26)	0.55 (0.26)	0.40 (0.19)
DNO SL	0.43 (0.36)	0.30 (0.19)	0.38 (0.43)	0.41 (0.32)	0.47 (0.30)	0.39 (0.33)

**Fig. 3:**
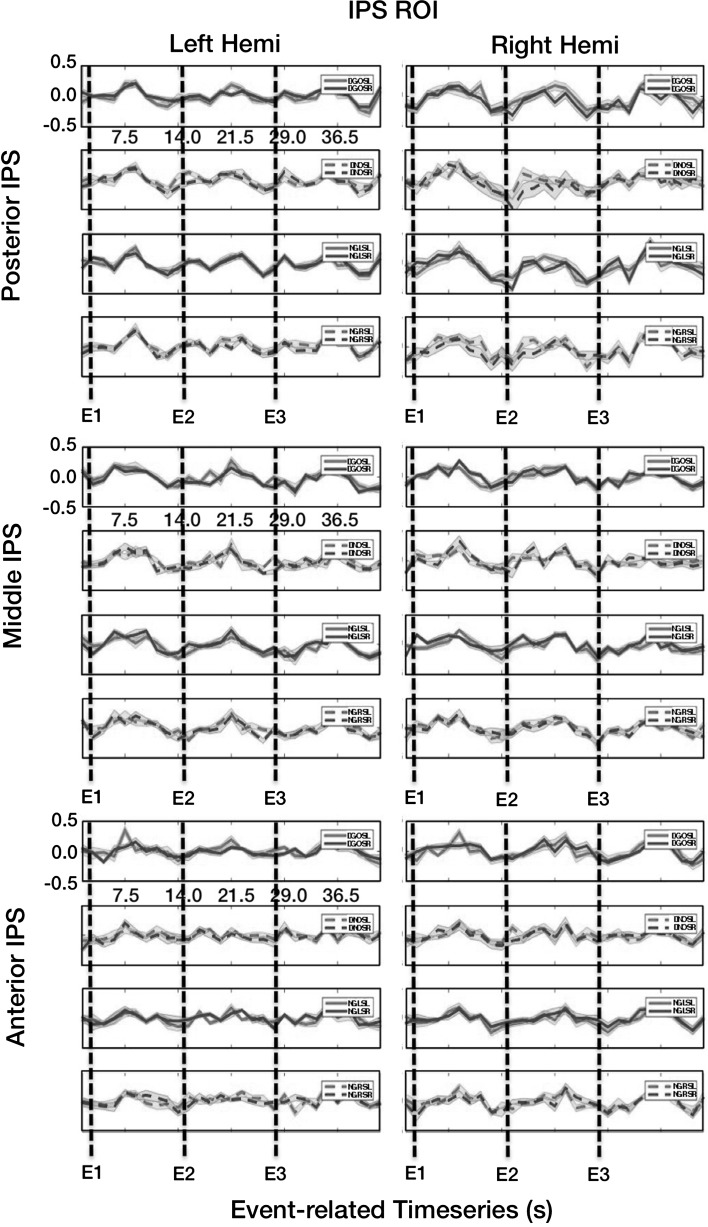
The slow event-related time courses separate by hemisphere, IPS zone and experimental condition. There are three discernible signal peaks in the activation time courses. These corresponded—following a shift to compensate for the hemodynamic lag—with the middle portion of each of the three epochs of interest (intention, attention and motor execution/withholding), and thus, these are not time-locked to the stimulus change and this observation discounts the argument that these represent purely sensory-driven responses. First, there is no difference between planning for an eye or point movement in all three IPS zones. Second, there is equally high activation amplitude for the attention epoch (or Epoch 2). Specifically, even when the movement had been cancelled, there is nevertheless, an fMRI-BOLD response that rivalled that of when the movement is to be executed. These two findings taken together provide for strong support that attention is coded to a relatively greater extent across the entire human IPS. *DGO* double go trial, *DNO* double no-go trial, *NGL* no-go left, *NGR* no-go right, *DGOSL* double go trial saccade left (and point right), *DGOSR* double go trial saccade right (or point left). The onset of the three epochs are demarcated via three *vertical lines* (for E1, E2 and E3)

Firstly, although we employed an event-related model that would allow for our fMRI-BOLD responses to take any possible waveform profile in the time domain, it is noteworthy that there nevertheless were three clear and discernible peaks in each of the responses (Fig. 1b), and each of these three peaks (Fig. 3) corresponded extremely well with our three functional epochs of interest [or intention, attention and motor execution/withhold epochs (Fig. 1)].

Each of the plots compares the responses for plan saccade left/plan point right [or saccade left (GL) or green peripheral circle left and saccade right (GR) or green peripheral circle right] for every condition of interest and is further separated by hemisphere. Notably also, the standard errors for each of these time courses (Fig. 3)—or lighter shaded regions above and below the mean time course values—were extremely small in amplitude and this gives confident that results were robust. It could thus be argued that there was little variance exhibited between the different participants in their fMRI-BOLD responses over time, and therefore, each participant showed “3 peak” modulation in each of the three IPS ROIs of interest.

For the third and final epoch, for the DGO as compared to the double no-go conditions, both conditions showed similar modulations in the fMRI-BOLD responses and in their peak fMRI-BOLD responses. This third and final peak (or Epoch 3) was comparable for the double no-go as compared to the either or both of the first two epochs. There was again a peak in response for Epoch 2 (the attention epoch) for double no-go (or no movement planned) that rivalled that for when DGO (both movements planned) was cued. Thirdly, during the first intention epoch, the saccade and point preparatory responses completely overlapped.

When considered collectively, these observations provide support for the idea that these were not purely sensory-induced responses—as during Epoch 3, only the fixation cue briefly changed from a crosshair to a circle, whereas in the first two epochs, the crosshair was subsequently flanked by two high-contrast and flashed peripheral targets—which would have presumably invoked a substantially greater sensory response (so, if this were sensory activation, then we would predict the highest activation during Epochs 1 and 2 and relatively lower activation during Epoch 3, and this would furthermore be time-locked to the stimulus change). Second, given that even when the movement command was cancelled during Epoch 2, the fMRI-BOLD responses remained high—and this observation suggests a robust attention component along the IPS. This argument is further confirmed by the observation that there was no difference in peak activity for eye as compared to point planning in Epoch 1. We would therefore argue that the present results provide further support for the idea of attention coding being relatively more prevalent throughout the healthy human IPS as compared to intention coding.

We then followed up these visual inspection observations with our statistical calculations, or via repeated-measures ANOVAs. Consistent with the above arguments, all of the main effects or interactions were non-significant for any of the comparisons of interest. Moreover, all of these comparisons exhibited *extremely* small partial eta-squared values, and these values indicate the strength (or in our case, the lack thereof) of the effects (all partial eta approached zero and for every condition examined). This further statistical detail assists to further rule out that our results cannot be attributed to a lack of sufficient statistical power (or had further participants been tested via fMRI, it is highly unlikely that our results would have changed).

First, there was no main effect of hemisphere and a negligible effect size *F*_(1,10)_ = 0.166, *p* = .692, *η*_*p*_^2^ = .016. There was also no main effect of the IPS zone (or ROI) examined, *F*_(2,20)_ = 0.429, *p* = .657, *η*_*p*_^2^ = .016. There was no main effect of epoch, *F*_(2,20)_ = 0.195, *p* = .824, *η*_*p*_^2^ = .016. Lastly, there was no main effect of condition, *F*_(7,70)_ = 1.71, *p* = .120, *η*_*p*_^2^ = .146.

We then examined the peak fMRI-BOLD signal changes for any interactions. Again, none of these were significant and all had extremely small or *negligible* effect sizes. Firstly, there was no hemisphere × IPS zone interaction, *F*_(2,20)_ = 1.329, *p* = .287, *η*_*p*_^2^ = .117. There was no interaction of hemisphere × epoch, *F*_(2,20)_ = 0.113, *p* = .894, *η*_*p*_^2^ = .011. There was no interaction of IPS zone × epoch, *F*_(4,40)_ = 0.613, *p* = .655, *η*_*p*_^2^ = .058. The hemisphere × condition interaction was non-significant, *F*_(7,70)_ = 1.650, *p* = .136, *η*_*p*_^2^ = .142. The IPS zone × condition interaction was non-significant, *F*_(14,140)_ = 0.632, *p* = .835, *η*_*p*_^2^ = .059, and the main effect of epoch × condition was non-significant, *F*_(14,140)_ = 1.020, *p* = .437, *η*_*p*_^2^ = .093. The statistical results are therefore highly consistent for both Fig. 3 and as compared the time courses to the response amplitudes in Tables 1, 2 and 3.

The three-way interactions also all produced null results—and despite the three modulations of the fMRI-BOLD responses, again, all complex interactions produced extremely negligible effect sizes. Firstly, a hemisphere × IPS zone × epoch interaction was non-significant, *F*_(4,40)_ = 1.664, *p* = .177, *η*_*p*_^2^ = .143. The hemisphere × IPS zone × condition interaction was non-significant, *F*_(14,140)_ = 1.452, *p* = .137, *η*_*p*_^2^ = .127. The three-way hemisphere × epoch × condition was non-significant, *F*_(14,140)_ = 0.996, *p* = .460, *η*_*p*_^2^ = .091, and also the IPS zone × epoch × condition, *F*_(28,280)_ = 0.828, *p* = .718, *η*_*p*_^2^ = .077. Finally, the four-way hemisphere × IPS zone × epoch × condition was non-significant and with an extremely negligible effect size, *F*_(28,280)_ = 0.643, *p* = .920, *η*_*p*_^2^ = .060. We therefore conclude that the human IPS preferentially codes for visual-spatial attention.

## Discussion

The present experiment directly compared two highly established paradigms—one for intention (Snyder et al. 1997) and a second for the identification of attention (Bisley and Goldberg 2003) coding—as utilized in the nonhuman primate brain via electrophysiology recordings. Rather than using electrophysiology, the present experiment employed event-related functional MRI and with a strict equidistant anatomical/functional-based parsing procedure of the IPS to contrast these two paradigms directly. In contrast to two of our three hypotheses, here it is reported that attention must play the relatively greater role across the IPS as compared to intention and based upon the following three consistent observations: (1) there was no difference between “intention” fMRI-BOLD signals when planning for saccade as compared to planning to point during Epoch 1, and (2) the double no-go (or the cancelled movement planning interval) still exhibited highly comparable levels of fMRI-BOLD response amplitudes as compared to the DGO (planning for both types of movement simultaneously) interval of Epoch 2, also emphasizing attention coding. Moreover, these enhancements were not time-locked to the stimulus presentation and therefore cannot be argued to represent sensory bursts. We therefore conclude that the present data are consistent with much earlier work that supports the argument that the IPS codes to a relatively greater degree for visual-spatial attention. We further argue that a purely sensory excitation argument can be ruled out, secondly, owing to the observation that Epoch 3 exhibited comparable levels of activation and yet there were no flashed peripheral targets presented during this time window. We are of the opinion that such fMRI-BOLD activity would have been highly diminished during Epoch 3—especially for the double no-go condition when the movement planning had been cancelled for a extended period of time—as compared to Epochs 1 and 2 when the high-contrast peripheral targets were flashed on the screen in the scanner.

The present data are therefore consistent with much of the extant human functional MRI literature on this same topic. For example, Astafiev et al. (2003) also reported comparable levels of recruitment and that this was independent of response demands or type of effector across the human IPS and these authors also examined eye and pointing movements—although these same authors did identify pointing-specific responses that were lateralized to the left hemisphere, but these same response were localized in the superior parietal lobule and precuneus. Liu et al. (2010) injected muscimol—a GABA_A_ antagonist—into different portions of the nonhuman primate lateral intraparietal area (LIP). These authors examined dorsal and ventral LIP separately and reported that such chemical-induced lesions of LIPv affected both saccades and attention (or a visual search task in their experiment). This is quite pertinent to the present results owing to our partitioning scheme, in which their nonhuman primate LIPv—which demonstrated no difference between attention and intention—would correspond most closely to the fundus of the sulcus. As noted in our “Methods” section, this was the area that pertained to our voxels (albeit in the human brain). Another experiment that examined prospective coding and mapped the activation onto a canonical brain showed that prospective coding was most predominate in the superior parietal lobule (Lindner et al. 2010). Finally, an event-related analysis that utilized multi-voxel pattern analysis (MVPA) also reported no differences in the signal amplitudes for saccades and whole-arm reaches (Gallivan et al. 2011a)—albeit this null result was restricted to the event-related analyses only.

A limitation that must be noted with regard to the present experimental design is that on 50 % of the trials the second epoch (or “attention” epoch) would reflect a pure motor plan.

It is necessary to note that there exist substantial differences between the present experimental protocol and that utilized by Bisley and Goldberg (2003): (1) we did not calculate psychophysical contrast thresholds, and (2) owing to the fact that we did not isolate receptive fields (RF), we cannot directly contrast cues presented either in or out of these RF. Moreover, the key finding of this earlier study was that at short stimulus onset asynchrony (SOA), the distractor decreased the contrast threshold, consistent with the attention argument.

Nevertheless, we are of the opinion that we do, in fact, isolate the effects of attention and for the following three reasons: (1) during Epoch 1, we analysed data in the left hemisphere for planned pointing with the right hand and these overlapped for future planned eye and hand movements. Owing to the transient nature of the flashed visual stimulus and the combined delay, this is consistent with attention, rather than early motor planning. For, if it was the case that it was early motor planning, then trials in which the pointing target appeared in the contralateral side of space (the right side of the screen), then we would predict enhanced fMRI-BOLD activity for those same trials for hand as compared to eye movements in the left hemisphere as compared to when a saccade target was place in the same location; (2) during the second epoch, all signals completely overlapped for GO and NOGO cues. This, again, is consistent with a substantial role in visual-spatial attention. For, if these same fMRI-BOLD signals were related to intentional early motor planning, then we would predict that these same signals would be attenuated on NOGO trials (and these were not); and (3) there were equivalent enhancements in the fMRI-BOLD response during Epoch 3—*even when the movement had been effectively cancelled for two successive epochs*. We therefore must conclude that the equivalent increases in response to MOVE or NOMOVE in Epoch 3 are owing to the appearance of the flashed central fixation cue only and that the intentional computations are effectively “swamped” by the attentional computational resources.

Perhaps the most difficult point to reconcile for these results is the observation that the parietal cortex has been successful used for brain–machine interface applications in the nonhuman primate (Andersen et al. 2010; Musallam et al. 2004). It is therefore difficult to argue that there does not exist an intentional role within voxel-based neuronal populations within the healthy human PPC also. Nevertheless, we do not feel that the present experiments are at odds with this important discovery. Rather, we conclude that the fMRI-BOLD response is simply relatively more sensitive to attention-related signals than to the intentional ones. In other words, visual-spatial attention must predominate within the human PPC as compared to intention-based signals. We can conclude this, given that we found a null effect for saccade as compared to point planning across the entire IPS zone of interest and that even when the movement was cancelled the responses still remained highly elevated during Epoch 2.

We should also duly note that it is likely to be the case that voxel-based functional MRI may not be spatially sensitive enough to the output layer neuronal projections (layer 5) of areas such as the parietal reach region that are known to be involved at least to some extent in reach planning (Snyder et al. 1997, 2000). Therefore, the fMRI responses in these same regions may be more specific to the afferent input and interneuron activity patterns, as demonstrated via combined electrophysiology and fMRI (Logothetis 2002; Logothetis et al. 2001). Nevertheless, one would suspect that given the modularity of the IPS and, in particular, that we examined the entire sulcus, there would still nevertheless exist strong afferents into the most anterior zone from the posterior nonhuman primate reach region, the area that we call human aIPS. Yet even here we did not observe a relatively greater intentional planning for hand movements, and moreover, this same area is well known to be involved in pre-shaping of the hand during grasping, in both the nonhuman (Sakata et al. 1995, 1998) and human primate (Cavina-Pratesi et al. 2013). As such, we were quite surprised to not find evidence of intentional point planning in the human aIPS. This study is therefore consistent with certain other fMRI studies that have demonstrated such a relatively strong role for visual-spatial attention throughout (or within certain areas) of human PPC.

It should also be noted that there exist limitations across the paradigms used by Snyder et al. (1997) and Bisley and Goldberg (2003) in the nonhuman primate for the present fMRI adaptation of these earlier experiments. Firstly, we only used only two possible target locations placed along the horizontal meridian, whereas Snyder and colleagues present targets at one of eight possible locations arranged around the start position. Second, we used “rotation-about-the-wrist” pointing movements, whereas this same experiment used whole are reaching to touch one of the eight targets on a particular trial and these reaching movements were executed relative to touching the centre of a touch panel. Moreover, in the Bisley and Goldberg (2003) study, the go/no-go probe always occurred at precisely the same location as the initial planning target—as these authors used receptive field-based electrophysiology. Moreover, these authors also incorporated a task-irrelevant distractor during the delay and used contrast thresholds. Finally, in both of the above studies, receptive fields were mapped and the data were sampled at a much higher temporal precision allowing for very short temporal epochs (in the range of 100 s of ms). Such key methodological differences make it possible that our paradigm is not directly comparable to these earlier studies. Second, and perhaps most importantly, given that the participants always planned movements to two possible cued locations—it could be argued that this led to a biasing of the present protocol towards visual-spatial attention for our “intention” epoch also.

One recent study also reported an absence in fMRI signal amplitude differences between planned movements, albeit in the realm of different types of object-directed grasp and reach movements (Gallivan et al. 2011b). Nevertheless, there did exist differences in the spatial activation localization across these two types of movements. Although our amplitude similarities are consistent with this earlier report, it should be noted we did not employ a precise spatial analysis that would have probed for spatially distinct subpopulations, such as multi-voxel pattern analysis in the reach planning domain (Filimon et al. 2014).

It is particularly noteworthy that an earlier study compared planning to reach and saccade using separate topographic-mapped IPS overlay zones (Levy et al. 2007). These authors also reported a null effect for the posterior to middle regions of the parietal lobule, and their results are therefore—for the most part—highly consistent with the present results. Moreover, consistent with this earlier study, we also reported null effects and similar amplitude changes in the fMRI-BOLD response (~0.8 % signal change for our data as compared to 1.0 % signal changes of this earlier study). Although our signal peaks typically ranged from 0.4 to 0.5 %, this factor must be effectively doubled in order to calculate our overall change as the oscillations in our study were negative going by approximately the same amount about the “0” point on the *y* axis—so our signal modulations were in the range of 0.8–1.0 % also.

There is one study in particular that must be rectified with the present results that did show a positive motor planning response for pointing along the medial aspect of the PPC (Connolly et al. 2003). It should be noted that there are inconsistencies perhaps owing to: (1) that our current ROIs were designed to capture the IPS and thus did extend as far into the medial precuneus parietal area 5 as this previous study that found evidence for point planning, and (2) this earlier study was one of the very first to employ computationally demanding rapid linear deconvolution in the realm of motor control. Owing to the very early implementation of this particular design strategy, the subsequently less precise linear deconvolution approach used at that time may be less reliable than current approaches that now incorporate double gamma functions (to monitor the consequent dip in the fMRI response post-enhancement and even their temporal derivatives in order to achieve a more optimal model fit). For these two reasons, our results are not necessary directly comparable to this earlier study.

It is critical to discuss the limitations of our null results and the potential limitations (or a potential for a false negative given our relatively small sample size). In a very recent and, in our opinion, exacting theoretical review article on this same topic, Vadillo et al. (2015) note that: “There is growing concern about the high rate of false positives within published studies. The literature contains far more significant findings (false positives) than it should. Research and/or journals are biased toward publishing significant results”. This statement represents, of course, the well-known file-drawer problem (Fanelli 2010; Ferguson and Heene 2012). Yet based upon our preceding discussion of the human fMRI literature on motor planning in the IPS—and although an entirely qualitative rather than a quantitative (or meta-analytic approach)—all of these human fMRI studies do report a null effect for motor planning along the IPS in particular. It should also be noted that certain studies incorporated relatively large sample sizes as compared to the present study—and yet obtained the very same experimental finding—no evidence for intention coding using fMRI along the human IPS.

Nevertheless, as these same authors state: “Null results are inherently ambiguous. Either the null hypothesis is true or there is insufficient evidence to reject it. A key determinant of the quality of an experiment is the number of trials on which its measurement is based … Studies conducted on larger samples are more likely to yield results that converge to the true effect size”. However, as these same authors also state: “Large and significant effect sizes are more likely to be obtained in low- than in high-powered experiments”. Again, the first point is an admitted weakness of the present findings. We have a small sample size and even for a ROI-based study (only 11 functional scans). Yet with regard to the latter point raised by these authors in particular, we can be at least reasonably confident in our data given: (1) there were *extremely small* residual error values observed for the mean fMRI-BOLD time courses; (2) the eta-squared effect sizes were *negligible or even approached perfect zero*; and (3) we utilized a well-established (or robust) paradigm for intention coding from the nonhuman primate literature. For all of these reasons, we are of the opinion that that the present results, although null, are valid and therefore are unlikely to represent a false-negative contribution to the literature. As a final point: these same authors note: “If an experiment yields a precise (narrow) confidence interval around zero, it is legitimate to conclude that the null hypothesis is supported by the data”. Again, although we discuss the literature *qualitatively* rather than quantitatively (or via meta-analysis), other fMRI studies show a null result for planning along the human IPS. Once again, the error values in the present data are extremely small in magnitude. Therefore, at least in our opinion, all of the above arguments lend credence that the confidence interval in a quantitative review of this area of research—if carried out—would also include zero for motor planning activity along the human IPS.

Another issue to point out is that we were surprised to observe that in the third epoch, the go versus no-go comparison had comparable levels of activation although no movement was required and we attribute this observation to a further attentional modulation. Nevertheless, previous research typically reports that the no-go response returns to baseline, e.g. Hester et al. (2004)—which we expected to occur as well—yet the activation showed an approximately Gaussian-shaped enhancement. As this response profile is similar to those observed in the other epochs, we attribute this yet another attentional modulation. Moreover, this is owing to the fixation cross changing to a circle after a relatively long intertrial interval. However admittedly intuitive, when practicing the task ourselves we noticed that this change after such a long interval invoked substantial attention resources. Third, this response was also not time-locked to the occurrence of the stimulus change and thus cannot be attributed to a sensory response. It is for all of the above reasons that we conclude that we observed ~equivalent responses in the third epoch across go and no-go trials.

In conclusion, the present study compared and contrasted directly in different temporal epochs two high-ranking electrophysiology paradigms (Bisley and Goldberg 2003; Snyder et al. 1997) using functional MRI. It is herein reported that similar to many other functional MRI studies that attention coding is relatively enhanced overall and throughout the entire IPS. That is, when compared and contrasted directly, there was no difference in signal amplitudes when preparing to point or saccade and, second, the observation that even when a movement plan is effectively cancelled the fMRI-BOLD response remains as high as when the movement is not cancelled (attention coding). Future studies should compare and contrast these very same paradigms using a paradigm suited to multi-voxel pattern analysis to determine whether or not distinct sub-regions such as those reported here preferentially encode saccade or point planning and/or to utilize even more ecologically valid (whole-arm) reach-and-grasp planning with kinematic variables recorded in the scanner environment and on a trial-by-trial basis.
